# Reply to “Comment on ‘*Pneumocystis jirovecii* Pneumonia in Patients Treated for Solid Organ Malignancy’ ”

**DOI:** 10.31486/toj.24.5047

**Published:** 2024

**Authors:** Ian Jackson, Raul Isern, Stephanie Jesina, Manasa Velagapudi, William Pruett

**Affiliations:** ^1^ Division of Pulmonary, Critical Care, and Sleep Medicine, Creighton University School of Medicine, Omaha, NE; ^2^ Division of Infectious Diseases, Creighton University School of Medicine, Omaha, NE; ^3^ Department of Internal Medicine, Creighton University School of Medicine, Omaha, NE

## TO THE EDITOR

We thank Khan and Ahmad^1^ for their interest in our case series, “*Pneumocystis jirovecii* Pneumonia in Patients Treated for Solid Organ Malignancy.”^2^

We agree that the increasing use of dose-dense chemotherapy regimens makes this clinical situation an important issue. We also agree with the recommendations outlined in their letter—namely that further study of the true incidence of *Pneumocystis jirovecii* pneumonia in this population is warranted and that raising clinician awareness of the heightened risk of *Pneumocystis jirovecii* pneumonia can help with early recognition and management.

On behalf of all the authors, please accept our appreciation for the letter.
